# Rehabilitation Oculomotor Screening Evaluation in Persons with Traumatic Brain Injury

**DOI:** 10.3390/jemr19040070

**Published:** 2026-07-02

**Authors:** Aimy Vadeboncoeur, Chelsey Lai Kwan, Ada Mocanu, Sarah Schipper, Olivia Taylor, Elizabeth Dannenbaum, Joyce Fung

**Affiliations:** 1 School of Physical and Occupational Therapy, McGill University, 3654 Prom Sir-William-Osler, Montreal, QC H3G 1Y5, Canada; aimy.vadeboncoeur@mail.mcgill.ca (A.V.); chelsey.laikwan@mail.mcgill.ca (C.L.K.); ada.mocanu@mail.mcgill.ca (A.M.); olivia.taylor2@mail.mcgill.ca (O.T.); 2 Jewish Rehabilitation Hospital Laval Site of Health Quebec and Research Site of the Montreal Center for Interdisciplinary Research in Rehabilitation (CRIR), Laval, QC H7V 1R2, Canada; edannenbaum_hjr@ssss.gouv.qc.ca

**Keywords:** oculomotor, eye movement, screening tool, traumatic brain injury, neurology, rehabilitation, functional assessment

## Abstract

**Background**: Many individuals with traumatic brain injuries (TBIs) exhibit oculomotor dysfunctions that impact their daily functioning. As current clinical screening tools are limited, we have created and pilot-tested the Rehabilitation Oculomotor Screening Evaluation (ROSE) previously in a small sample of people with acquired brain injuries and neurotypical participants. The current study aims to validate ROSE in persons with TBI, focusing on mild TBI (mTBI). **Methods**: Participants with TBI (*n* = 25) completed different clinical scales, including ROSE, Sensory Organization Test (SOT) for standing balance, Reintegration to Normal Living Index (RNLI), Timed Up and Go (TUG) for mobility, and a visual analogue scale for the subjective perception of visual vertigo. Neurotypical individuals (*n* = 24) who were age- and sex-matched completed only ROSE. **Results**: The group with mTBI (*n* = 18) had significantly higher ROSE scores compared to the neurotypical group, with a large effect size. Significant correlation was found between ROSE and RNLI scores, but not with other clinical outcomes. **Conclusions**: Significant between-group difference in ROSE scores and their association with RNLI scores suggest that ROSE is a valid tool in detecting oculomotor dysfunction in TBI. Future studies should continue the validation of ROSE in other TBI and neurologic populations and in larger sample sizes.

## 1. Introduction

Oculomotor dysfunctions (OMDs) are eye movement disorders affecting extraocular muscle control and provoking diverse symptoms depending on the underlying neurological conditions. Individuals with OMD may have limited tolerance to perform activities of daily living, occupation, and hobbies [1,2]. After having a traumatic brain injury (TBI), people may have OMDs, interfering with the ability to participate in various activities, including reading, driving, and engaging in busy environments (e.g., grocery stores, public transportation, etc.) [3,4]. TBI is defined as a change in brain function, or other evidence of brain pathology, resulting from an external force [5]. The severity of injury is classified as mild, moderate, or severe by the Glasgow Coma Scale upon acute care admission [3]. Oculomotor control relies on a vast neural network involving cortical and subcortical regions that are frequently disrupted following TBI, leading to deficits in saccades, smooth pursuit, and gaze stability [6]. As such, individuals with TBI may have varying symptoms and possibly unnoticed and untreated OMD. These impairments can serve as sensitive indicators or underlying neurological dysfunction [6]. However, despite increasing use of vision-based assessments in concussion, their role as validated clinical screening tools remains incompletely established [7]. Thus, a valid screening tool is needed to detect OMD and to support effective TBI intervention in rehabilitation settings [8,9].

We have previously created the Rehabilitation Oculomotor Screening Evaluation (ROSE) as a standardized clinical tool to screen for OMD in acquired brain injuries (ABIs). ROSE takes on average 15–25 min to complete, ranking the quality of eye movements in different oculomotor tasks, as well as symptom provocation via a visual analogue scale (VAS). The oculomotor tasks include 4 categories: (1) Smooth pursuit and vergence; (2) Saccades; (3) Eye cover tests and gaze fixation; and (4) Vestibulo-ocular reflex (VOR) and cancellation (cVOR). These domains were selected based on prior literature highlighting their relevance in detecting vestibulo-ocular dysfunction following mTBI and their inclusion in established screening tools such as the Vestibular/Ocular Motor Screening (VOMS) [10]. The scoring for each item in each category is ranked numerically as 0 (normal), 1 (abnormal), or 2 (severely abnormal). The summed score thus ranges from zero (no deficit) to 48 (maximum deficit) for a total of 16 items in 4 categories.

In the pilot study [8], ROSE was administered on 10 participants with ABI that included TBI and cerebrovascular accident (CVA), as well as 10 sex- and age-matched neurotypical participants. We found significant differences between ABI and neurotypical participants, with marked symptom provocation found mainly in TBI participants. We concluded that ROSE is potentially a comprehensive and standardized tool to detect OMD [8]. The current study was undertaken to assess OMD with ROSE, focusing on TBI in the rehabilitation setting.

The first objective of this study was to assess the construct validity of ROSE by comparing the total summed scores between TBI and neurotypical individuals. The second objective was to assess the relationships between ROSE and rehabilitation outcome measures of balance, social integration, mobility, and self-reported scores of visual vertigo.

## 2. Materials and Methods

### 2.1. Participants

Over a 2-month period, 25 participants with a diagnosis of TBI were assessed from the traumatology and vestibular programmes at the Jewish Rehabilitation Hospital (JRH), a research site of the Centre for Interdisciplinary Rehabilitation (CRIR) of Greater Montreal and a rehabilitation teaching hospital of McGill University in Quebec, Canada. Participants were matched with 25 healthy controls by age and biological sex using convenience sampling. Participants must be aged between 18 and 66 Years old. Exclusion criteria included: (i) severe ocular diseases (i.e., glaucoma, diabetic retinopathy, macular degeneration, etc.); (ii) inability to comprehend or speak English and/or French; (iii) inability to actively participate and follow instructions; (iv) not wearing corrective lenses as required during testing; and (v) neurological condition other than TBI. Information about TBI severity was retrieved from medical charts, except for one self-report, since the participant did not consent for their medical chart to be accessed. Eligibility and exclusion criteria for neurotypical participants were identical to those with TBI. Ethics approval was granted by the CRIR ethics review board. Written informed consent was obtained from all eligible participants prior to study participation.

### 2.2. Administration and Assessment of ROSE

Minor clerical changes were made to the original ROSE to improve clarity and succinct application of the tool. Paired evaluators administered the ROSE tool, one as the tester and the other as an observer. The tester administered the ROSE tool and marked the score. The observer offered support and assisted with test logistics, including documenting total assessment time and preparing equipment. Both the observer and the tester were occupational therapy or physical therapy students (AV, CLK, AM, SS, OT) trained by the creators of the ROSE tool (ED, JF), and they followed the instructions document (see Supplementary File S2). ROSE testing took place in an enclosed clinical setting devoid of windows to limit visual and auditory stimuli. Behind the tester, a plain white background was used to limit any visual distractions. The tester used a printed script (English or French) to minimize inter-tester variability and standardize the instructions provided to participants. Although evaluators followed a standardized administration protocol, inter-rater reliability was not assessed in the present study, as it was not an explicit objective of the present study.

During screening, participants were seated at a desk in a standardized position with the head maintained in a neutral, stabilized posture throughout testing. Symptoms were monitored continuously, and participants also reported symptom severity over the preceding 24 h. The initial assessment consisted of observational screening, during which the examiner noted the presence of head tilt, head tremor, ptosis, eyelid retraction, lid fasciculations, and pupillary responses (PERRLA) using a handheld light source.

For smooth pursuit assessment, a fixation target was positioned approximately 40 cm from the participant’s nasion using a ruler for standardization. Participants were instructed to maintain head stability while visually tracking the target as it moved slowly up to 45° from midline in horizontal, vertical, and vergence planes. Each movement cycle was performed twice in each direction. The examiner recorded the presence of corrective saccades, asymmetry, or impaired tracking.

Vergence was assessed using a target moved along a 30 cm ruler positioned at the participant’s midline, approximately 2 cm above the nasion. The target was advanced slowly toward the participant until diplopia was reported or outward deviation of one eye was observed. The near point of convergence and recovery distance were recorded. Three trials were conducted. If diplopia was not elicited, the test was excluded from scoring. Excessive blinking was also documented.

Saccadic function was evaluated by instructing participants to alternate gaze between two targets positioned on a ruler at a distance of 40 cm (15 cm for vergence-related trials) for 8 s. The number of completed cycles was recorded. Movement accuracy and symmetry were qualitatively assessed, including the presence of hypometria, hypermetria, dysmetria, apraxia, ocular flutter, or overshoot.

The fixation and alignment category included three subtests. For fixation in eight gaze directions, participants were asked to fix their gaze on a stationary target (examiner’s fingertip) positioned approximately 40 cm away at 45° in vertical, horizontal, and diagonal directions for at least 4 s in each direction. Gaze stability, loss of fixation, and nystagmus were recorded. The cover test involved occluding one eye for 2 s while the participant fixated on the examiner’s nose; corrective movements in the uncovered eye were noted. In the alternate cover-uncover test, each eye was occluded for at least 1 s in an alternating sequence for four cycles, with observation of refixation movements upon uncovering.

Vestibulo-ocular reflex (VOR) and cancellation (cVOR) were assessed using two tasks. Participants began with cVOR as they sat with feet flat on the floor, arms extended, and hands clasped with thumbs up. While maintaining visual fixation on their thumbs, the examiner rotated the participant’s trunk through approximately 80° at a metronome-paced frequency of 50 beats per minute for five cycles. For VOR testing, participants fixed their gaze on a stationary visual target (printed letter “E”, size 12) positioned at eye level with approximately 20° of neck flexion. The examiner passively moved the participant’s head horizontally and vertically through a range of approximately 25–30° at 120 beats per minute for five cycles while fixation was maintained. The full instrument, scoring sheet, and detailed administration instructions are provided in Supplementary Files S1 and S2.

### 2.3. Other Clinical Outcome Measures

Only participants in the TBI group went through testing with other relevant rehabilitation outcome measures. The Reintegration to Normal Living Index (RNLI) [11] and the Visual Vertigo VAS [12] were administered in the same dedicated setting as for the ROSE tool. Outcomes of mobility and balance, such as the Timed Up and Go (TUG) [13] and Sensory Organization Test (SOT) [14], were administered in the usual clinical setting.

### 2.4. Data Analysis

Descriptive statistics were used to characterize the groups (mild, moderate, and severe TBIs vs. no TBI), followed by the Shapiro–Wilk test to determine normality in the outcome distribution. Due to the small number of participants recruited in the moderate and severe groups and the normal distribution of ROSE in the mild (mTBI) group, the comparison was focused between mTBI and age- and sex-matched controls using an independent sample *t*-test. In addition, the Pearson *r* correlation tests were used to determine any significant correlation of the ROSE total scores with RNLI total scores, Visual Vertigo VAS total scores, TUG total scores, and SOT composite scores. A *p*-value of less than 0.05 was accepted as significant.

Based on the proposed construct underlying ROSE, we hypothesized that total ROSE scores would effectively differentiate between neurotypical (healthy control) individuals and those with mild, moderate, or severe TBI, with lower scores expected in the healthy controls compared to mTBI individuals. In the latter group, inverse correlations were expected between ROSE and SOT and RNLI scores, whereas positive correlations were expected between ROSE and TUG performance.

## 3. Results

### 3.1. Participant Characteristics

Fifty-one persons with TBI who were discharged or undergoing rehabilitation at the JRH were contacted by phone, of whom 25 declined, one was a no-show, and 25 were successfully tested. They included 11 males (M) and 14 females (F), with a mean age of 47.6 (±11.3) years old. The TBI severity levels were: mild (*n* = 18), moderate (*n* = 4), and severe (*n* = 3). In the control group, one participant had abnormal eye movements detected in the saccades and VOR/cVOR categories. The participant was referred to a vestibular therapist for further examination, but declined. This participant was excluded from the control group. The TBI group was thus matched with 24 controls with a mean age of 45.5 (±11.2). All participants could self-initiate mobilization, although one participant did not have their assistive device at the time of testing; the TUG test was not administered.

The analysis initially included the entire TBI group (*n* = 25), but ultimately focused primarily on the large subgroup of mTBI (*n* = 18). The mTBI group (5 M/13 F) had a mean age of 47.2 (±11.5). The control group in comparison was matched by age (47.7 ± 10.1) and sex (5 M/13 F). In the mTBI group, 61% (11/18) had one incidence of TBI; 67% (12/18) were in active rehabilitation, and 67% (12/18) had their TBI more than one year prior to testing (see Table 1). Given that 67% of participants were more than one year post-injury, the cohort may more closely represent individuals with persistent post-concussion symptoms rather than the general mTBI population. Due to the limited sample size, stratified analyses by time since injury were not feasible.

### 3.2. Group Distribution and Comparisons

**Figure 1:**
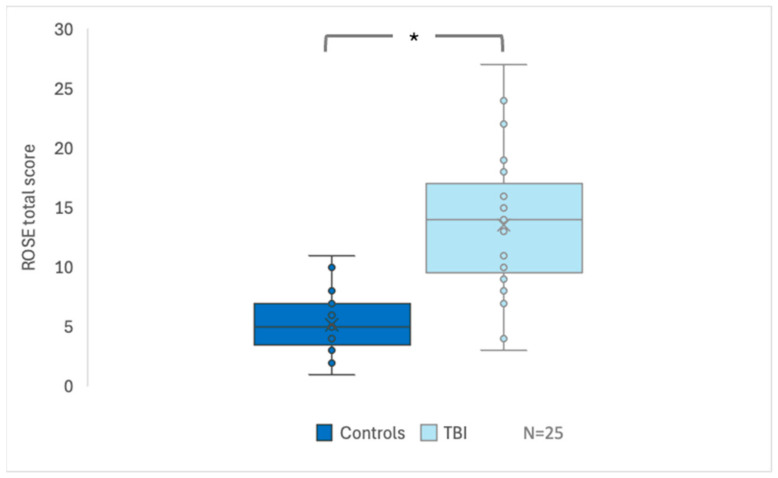
Between-group comparisons of ROSE scores in TBI.

The ROSE total score was found to be normally distributed for the entire TBI group (*n* = 25) and control groups (*n* = 24), as well as in the mTBI group (*n* = 18) (W = 0.98, *p* = 0.91; W = 0.97, *p* = 0.66) and the sex- an age-matched controls (*n* = 18) (W = 0.97, *p* = 0.81; W = 0.96, *p* = 0.63). Descriptive statistics with box plots were used to summarize the data, and independent sample *t*-tests were applied for comparisons (Figure 1).

The ROSE VAS total score was not normally distributed for both the TBI and control groups (W = 0.92, *p* = 0.04; W = 0.67, *p* < 0.01). Consequently, descriptive statistics using means and standard deviations were reported.

The RNLI was normally distributed (TBI: W = 0.98, *p* = 0.78; mTBI: W = 0.97, *p* = 0.84). Similarly, visual vertigo VAS scores were also normally distributed (TBI: W = 0.93, *p* = 0.10; mTBI: W = 0.94, *p* = 0.29). The TUG scores were normally distributed only for the mTBI group (W = 0.94, *p* = 0.30). Linear regressions were then applied to examine the correlations between ROSE and the normally distributed clinical outcomes.

After removing outliers and incomplete scores in the SOT composite scores (due to falls or participant withdrawals), normal distributions were found in both groups (TBI: *n* = 17; W = 0.99, *p* = 0.99; mTBI: *n* = 11; W = 0.96, *p* = 0.73). Linear regressions were then applied, and data were summarized using means and standard deviations.

### 3.3. Comparison of the ROSE Scores Between TBI and Control Groups

**Figure 2:**
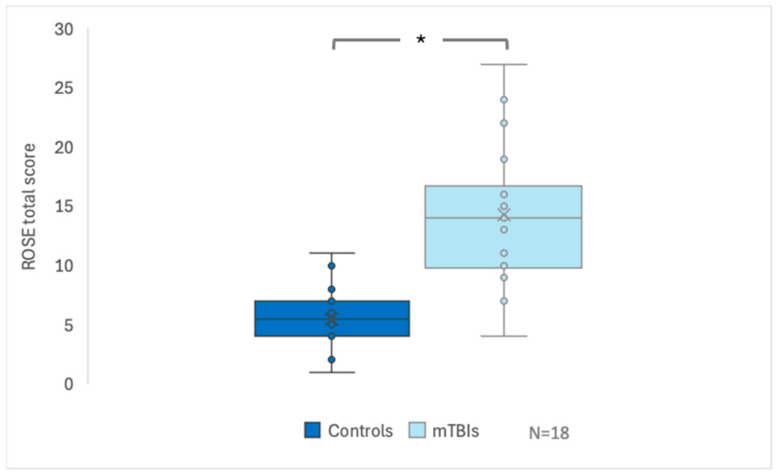
Between-group comparisons of ROSE scores in mTBI.

As illustrated in the box plots in Figure 2, the TBI group showed significantly higher scores compared to the control group (t(47) = 6.437, *p* < 0.001), with a large effect size (Cohen’s d = 1.84). Similarly, in a subgroup analysis of mTBIs and controls, the mTBI group also had significantly higher scores (t(34) = 5.667, *p* < 0.001) and a large effect size (Cohen’s d = 1.89) (Figure 2).

**Figure 3:**
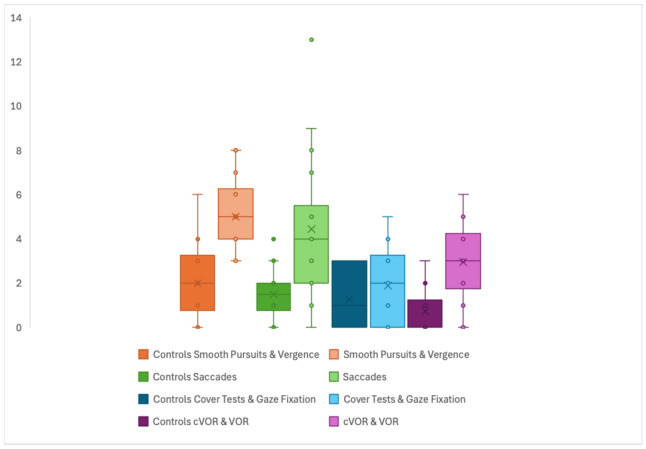
Between-group comparisons of subtest ROSE scores.

As illustrated in the box plots in Figure 3, the mTBI group had a mean score of 5.00 (±1.61), while the control group scored 2.00 (±1.71) for the SPV. The mTBI group’s mean score for SC was 4.44 (±3.24), while the control group scored 1.50 (±1.15). CTGF had a mean score of 1.89 (±1.64) for the mTBI group, compared to 1.28 (±1.32) for the control group. Finally, the mTBI group scored 2.94 (±1.80) for VORs, and the control group scored 0.72 (±0.96).

### 3.4. Association with Clinical Outcomes of Social Integration, Balance, Mobility, and Dizziness

**Figure 4:**
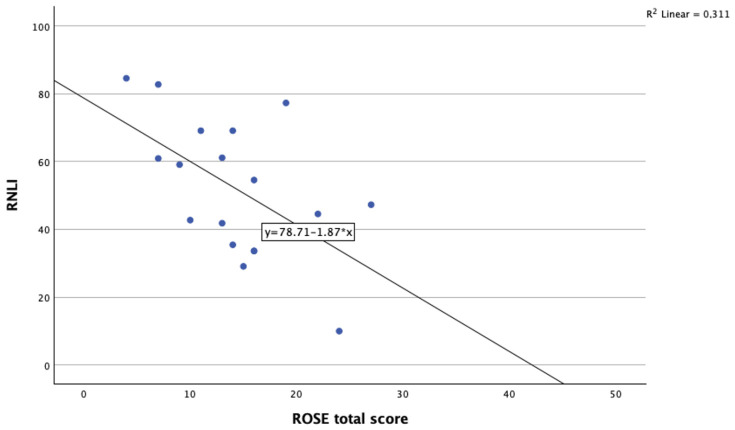
Significant correlation between ROSE and social participation (RNLI).

The simple linear regression in Figure 4 showed a significant negative association of ROSE and RNLI scores in mTBI participants, accounting for 31% of the variance (F(1,16) = 7.229, *p* = 0.02). Note that a high RNLI score indicates an increased level of independence, whereas a high ROSE score signifies OMD.

The associations between the ROSE and the other outcome measures were not statistically significant (TUG (F(1,16) = 2.971, *p* = 0.10), SOT composite (F(1,9) = 4.048, *p* = 0.09), and Visual Vertigo VAS (F(1,15) = 4.200, *p* = 0.06)). The clinical tests used for outcome measures in this study did not have a significant correlation with the ROSE total score, and thus, the associations are inconclusive with the current limited sample size (adjusted R^2^ values of 0.16, 0.31, and 0.22, respectively).

### 3.5. Symptom Provocation

The mTBI group had significantly higher total ROSE VAS score (M = 47.17, SD = 11.53) compared to the control group (M = 2.20, SD = 3.64), t(34) = 5.112, *p* < 0.001), with a large effect size (Cohen’s d = 1.70).

**Figure 5:**
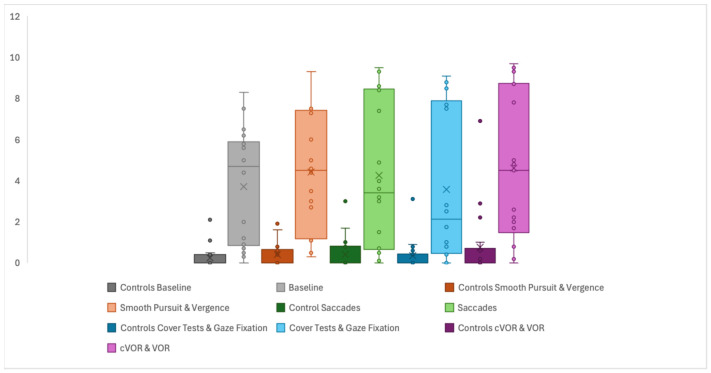
Symptoms provoked (VAS) in different ROSE categories in mTBI.

The box plots in Figure 5 shows the ROSE VAS subtest scores for the mTBI and control groups. At baseline, the mTBI group had a mean VAS score of 3.72 (±2.74), while the control group scored 0.26 (±0.54). The symptoms carried over from baseline to each category of eye movement testing in ROSE in participants with mTBI, but were not significantly higher than the baseline scores. A significant relationship was also found between the ROSE total and ROSE VAS total scores in mTBI (F(1,16) = 7.192, *p* = 0.02), accounting for 31% of the variance.

**Figure 6:**
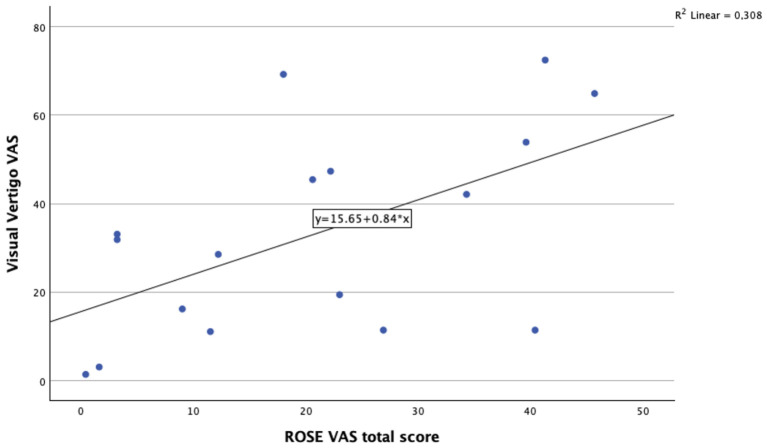
Correlation of Visual Vertigo VAS with ROSE VAS in mTBI.

These symptoms can be partially explained by the subjective perception of visual vertigo, as shown by the significant association between the ROSE VAS with the visual vertigo VAS in mTBI (F(1,15) = 6.663, *p* = 0.02), accounting for 31% of the variance (Figure 6).

## 4. Discussion

Our main finding reveals that ROSE can be used to detect OMD in people with TBI, as indicated by the significantly higher mean total ROSE scores and ROSE VAS scores in the TBI and mTBI groups compared to controls, demonstrating strong construct validity. Since there were limited numbers of moderate and severe TBI participants in the study, the analysis focused on the mTBI group, and the discussion will primarily address findings related to this subgroup. These findings align with prior work showing that multidimensional oculomotor measures can sensitively detect TBI-related dysfunction [6]. The large effect size (Cohen’s d = 1.84) demonstrates that our results have real-life relevance and informs sample size estimations for future studies to validate the ROSE tool in moderate and severe TBI (*n* = 20–25 for each severity category).

The observed pattern of correlations was generally consistent with our a priori hypotheses, with a significant association observed between ROSE and RNLI, suggesting that difficulties in social reintegration in mTBI are likely related to OMD. However, no significant association was found between other clinical outcomes of mobility and balance (TUG and SOT), suggesting that conventional rehabilitation measures may not evaluate the same constructs as ROSE. The non-significant associations observed with TUG, SOT, and Visual Vertigo VAS should be interpreted cautiously, as borderline *p*-values and meaningful effect sizes may reflect inadequate statistical power rather than the true absence of association. It is plausible that TUG is not a sensitive measure for changes in mobility due to OMD. TUG was originally created for a geriatric population [13]. The SOT was chosen as it calculates precise variations in standing balance based on sway measurements and was accessible at the clinical site. A limitation of the SOT in the current study is that it generated incomplete data for multiple participants due to attrition related to its specific testing parameters. The SOT testing paradigm was not well tolerated by some participants with limited standing endurance and comorbidities such as post-traumatic stress disorder, pain, and/or reduced endurance. Moreover, it is possible that people with mTBI may not have the insight to respond accurately to self-reported measures (VVAS) due to cognitive changes. In fact, impaired self-awareness has been reported across TBI subtypes [15]. In our study, this may indicate that responses to prompts result from denial or unrealistic assessment of abilities affected by the experience of dizziness. Finally, it is important to consider that a higher score on the ROSE does not necessarily correlate with more challenges in other areas, such as balance or mobility. The impact on these constructs and their connection to OMD is influenced by the specific region of the brain affected by the mTBI and any previous TBIs, which speaks to the complexity of research in this area, as well as the way the constructs are measured.

The current study cannot determine any association of OMD with the severity of TBI, due to the limited number of participants with moderate and severe TBI. The imbalanced ratio of M/F participants (5/13) with mTBI reflects the realistic conditions of sampling in a 2-month time frame. A longer recruitment period may allow for recruitment of a larger sample of TBI participants with a more balanced sex and TBI severity distribution. Furthermore, because most participants had sustained their TBI several months prior to assessment, ROSE scores may have been influenced by spontaneous recovery or compensatory oculomotor adaptations, potentially reducing the sensitivity of the screening tool. It is plausible that ROSE outcomes may differ across acute, subacute, and chronic recovery stages.

We have demonstrated that symptoms provoked during ROSE administration (ROSE VAS) were related to the ROSE total score itself in mTBI. In other words, a high score on OMD can potentially predict higher symptom provocation. However, a methodological limitation is that all ROSE subtests were administered in a fixed order. As a result, we are unable to confirm the extent to which individual subtests provoked the most symptoms, as observed increases in symptoms may reflect order effects. Worsening symptoms could be attributed to other factors, such as cumulative effects and fatigue. Future research should randomize subtest order to explore these relationships. The association between the ROSE VAS and the Visual Vertigo VAS suggests that mTBI participants’ perception of visual vertigo symptoms may be attributed to OMD-related symptom provocation.

Although a one-hour time slot was reserved for each participant to accommodate the completion of multiple assessments, most participants completed the evaluation in less time. Nevertheless, the length of the testing session may have contributed to sensory fatigue and symptom provocation, potentially influencing participant performance and subsequent ROSE scores. Additional factors include a perturbed field of vision with multiple clinicians simultaneously assessing eye movement, as well as neck pain and limited neck movement for VORs.

For future studies, the layout of the ROSE tool was updated to enhance clarity based on observations gathered during this study; note that the content remained the same. The ROSE tool was shortened from 5 to 4 pages by regrouping testing items and removing the VAS scoring from under each subtest. Instead, smiley-face icons were added near the scoring section of every VAS-scored item to remind clinicians to refer to the separate VAS scoring page. This reduces ambiguity for the clinician and participant when testing VAS and prevents the participant from seeing their ROSE scores mid-assessment. Furthermore, the screening for vergence was given its own subtest score since we observed a distinct score difference between smooth pursuit and vergence.

Eye fixation and eye cover/uncover tests do not demonstrate a difference in mean scores between mTBI participants and control participants; however, it is unknown whether there would be a difference in subtest scores within moderate and severe TBI groups. Previous testing of the ROSE included participants with a CVA diagnosis [8], in which some cases noted visual neglect. Neglect was not apparent nor flagged in the TBI participants during this study. We suspect that future studies sampling participants with CVA and more severe TBI will note differences in the gaze fixation and cover test subtests alongside observations of visual neglect [16,17,18,19]. Thus, we added a neglect check box in the notes section of the 8-direction gaze fixation in the updated ROSE tool. Additionally, during the TBI group testing, excessive blinking was observed in some participants, which may be indicative of fixation difficulty [20,21] and may be a symptom of photosensitivity following TBI [22]. Adding a score of 0–2 due to excessive blinking would increase the total ROSE score to 50 (See Supplementary File S1).

A current limitation of the ROSE tool is the lack of psychometric properties, especially inter-rater variability related to subjective evaluation of saccades in certain subtests. Distinguishing between categories may be influenced by the evaluator’s experience, perceptual threshold, and the absence of eye-tracking instrumentation. In addition, the smooth pursuit protocol relies on manual target movement, which may also introduce variability in stimulus velocity across examiners. The use of pacing aids such as a metronome or a digital target system may help reduce this variability. Future studies should evaluate reproducibility to further support the reliability of the tool.

With a large effect size shown by Cohen’s d comparing ROSE total scores between mTBI and controls, this study demonstrates that a minimum of 18 mTBI participants is sufficient to detect differences. However, we recommend using a sample of 20–25 participants per TBI severity group to improve construct validity. Considering the limitations of the TUG, future research may consider an alternative mobility assessment focused on quality of movement or endurance in adults, such as the Six-Minute Walk Test. Similarly, future research may use a more affordable and inclusive assessment tool, like the Clinical Test of Sensory Interaction on Balance (foam and dome), in place of the SOT to determine correlations between the ROSE scoring and balance endurance.

This study’s cross-sectional design limits causal interpretation of the observed associations. Although OMD could be associated with functional outcomes, the directionality of these relationships cannot be determined. Longitudinal studies are needed to clarify whether oculomotor impairments contribute to functional limitations over time. Establishing norms for various ages and populations should be considered for the deployment of ROSE as an OMD clinical screening tool. Throughout the study, results were shared with the treating physiotherapist or occupational therapist for patients who consented. Anecdotally, these clinicians noted that this information was helpful in planning treatment. In future studies, measuring the clinical utility of this information for clinicians treating people with TBI can be considered.

## 5. Conclusions

We have developed a new clinical tool, ROSE, that can be used to screen OMD in people with TBI. Building on a previous pilot study [8] to assess OMD in ABI, this study shows the utility of ROSE in screening OMD in individuals with TBI, as shown by the significant between-group differences in ROSE total scores of TBI and control participants. In addition, social participation or reintegration to normal living is significantly associated with OMD measured with ROSE, especially for participants with mild TBI. The current study shows evidence of the construct and the functional implications of ROSE as an outcome measure for OMD in mTBI.

## Figures and Tables

**Table 1:** Characteristics of the TBI group.

	TBI Group			
	Total	Mild	Moderate	Severe
	*n* = 25	*n* = 18	*n* = 4	*n* = 3
Mechanism of Injury				
Transport accident	11	6	2	3
Falls	6	4	2	0
Assault	3	3	0	0
Unintentionally struck by or against an object	5	5	0	0
Number of TBI episodes				
Only 1	15	11	2	2
≥2	6	6	0	0
Unknown	4	1	2	1
Active rehabilitation	15	12	1	2
Months since accident				
0–6	3	1	0	2
7–12	6	5	1	0
>12	16	12	3	1

## Data Availability

The original contributions presented in this study are included in the article. Further inquiries can be directed to the corresponding author.

## Supplementary Materials

The following supporting information can be downloaded at: https://www.mdpi.com/article/10.3390/jemr19040070/s1, File S1: ROSE score sheet; File S2: ROSE instructions.

## Abbreviations

The following abbreviations are used in this manuscript:

ABI	Acquired brain injuries
CVA	Cerebrovascular accidents
OMD	Oculomotor dysfunction
RNLI	Reintegration to Normal Living Index
ROSE	Rehabilitation Oculomotor Screening Evaluation
SOT	Sensory Organization Test
TBI	Traumatic brain injury
mTBI	Mild traumatic brain injury
TUG	Timed Up and Go
VOR	Vestibulo-ocular reflex
cVOR	Cancellation vestibulo-ocular reflex
VAS	Visual analogue scale
