# Supplementary material for: Rehabilitation Oculomotor Screening Evaluation in Persons with Traumatic Brain Injury
Source: J Eye Mov Res. 2026 Jul 2;19(4):70. doi: 10.3390/jemr19040070 (PMC13398043; doi:10.3390/jemr19040070)
Supplement: Supplementary file 1 [file jemr-19-00070-s001.zip › Supplementary File S1_ROSE score sheet.pdf]

2024 ROSE STUDY - GENERAL SCORING SHEET
Glasses: N / Y:
Corrected visual acuity (x/20):
Double Vision: N / Y: Horizontal/Vertical
Eye Pathology:

Patient name:

Date & Time:

*Note: ☻ = reminder to fill out VAS for symptoms

1) Observations

**All within normal limits** ☐			
Head tilt/turn		☐ Small (<10 degrees)	☐ Large (>10 degrees)
Head Tremor		☐ Small	☐ Large
Slow light reflex (PERRLA), or asymmetry ☐	No light reflex (PERRLA)	(L) ☐	(R) ☐
Mild ptosis/retraction/lid fasciculation		(L) ☐	(R) ☐
Severe ptosis/retraction/lid fasciculation		(L) ☐	(R) ☐

Additional Notes

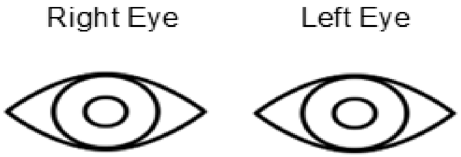

☻

Symptoms Rating in the last 24hrs:

Dizziness Nausea Headache

Fogginess Eye pain

Other:

2) Smooth Pursuits

**Movement Quality**		Horizontal	Vertical
Small amplitude: ~2-5mm	No jerkiness/ catch-up saccades **OR** less than 2 saccades	0	0
Large amplitude: >5mm	Small amplitude **OR** 3-5 saccades	1	1
	Large amplitude **OR** >5 saccades	2	2

**Symmetry of eye movements**		Horizontal	Vertical	Vergence
	Symmetrical	0	0	0
	Minor asymmetry	1	1	1
	Major asymmetry	2	2	2

**Smooth Pursuits Total Score**	**/10**
**Excessive blinking of the eyes**	(Y) ☐ (N) ☐

Notes:

*Mark the location of the saccade(s) during smooth pursuits (i.e., Horizontal, Vertical or Vergence)

2024 ROSE STUDY - GENERAL SCORING SHEET

Patient name:

Date & Time:

3) Vergence

Trial 1		Trial 2		Trial 3		Longest Distance	
NPC	Recovery	NPC	Recovery	NPC	Recovery	NPC	Recovery Δ
cm	cm	cm	cm	cm	cm	cm	cm

**NPC Score**<br>*NPC= Near point od convergence	≤ 5.0cm of the nasion	0
	>5.0cm & <10cm of the nasion	1
	≥10cm of the nasion	2
**Recovery Δ**	≤ 7.0cm	0
	>7.0 & <12 cm	1
	≥12 cm	2
	**Vergence Total Score**	**/4**
	**Excessive blinking of the eyes**	(Y) □ (N) □

Notes:

4) Saccades

	# of saccades:	Horizontal #:	Vertical #:	Vergence
**Number of Saccadic Cycles** *(there & back = 1 cycle)*	>7	0	0	0
	4-7	1	1	1
	<4	2	2	2

		Horizontal	Vertical
**Accuracy**	Accurate	0	0
	Mild under/overshoot	1	1
	Severe under/overshoot	2	2

		Horizontal	Vertical	Vergence
**Symmetry of mvts**	Symmetrical	0	0	0
	Mild asymmetry	1	1	1
	Severe asymmetry	2	2	2

**Saccades Total Score**	**/16**
**Excessive blinking of the eyes**	(Y) □ (N) □

Notes:

2024 ROSE STUDY - GENERAL SCORING SHEET

Patient name:

Date & Time:

5) Gaze Fixation + Cover Tests

Fixation in 8 Gaze Directions

		L	R
**Fixation**	Stable fixation in 8 gaze directions. No signs of gaze induced nystagmus or drift. Able to maintain fixation in end of range gaze position for ≥ 4s.	0	0
	Gaze induced nystagmus and/or drift observed in 1 direction.	1	1
	Gaze induced nystagmus and/or drift observed in >1 directions or not end of range	2	2

Notes:

Neglect: (Y) □ (N) □

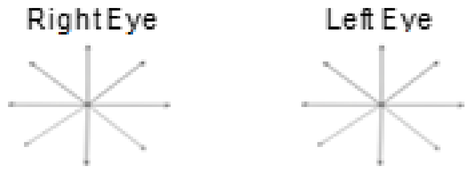

Eye Cover Test

		Left	Right
**Eye Movement**<br>*Score the eye that is* ***uncovered***	Uncovered eye remains on target (i.e., no correction)	0	0
	Uncovered eye moves outwards inwards	1	1
	Uncovered eye moves upwards downwards	2	2

Alternate Cover-Uncover Test

		Left	Right
**Direction of Deviation**	Eyes remain on the target	0	0
	Uncovered eye moves **mainly** □ outwards □ inwards	1	1
	Uncovered eye moves □ upwards □ downwards	2	2
	**Total Gaze Fixation + Cover Tests Score**	**/12**	
	**Excessive blinking of the eyes**	(Y) □	(N) □

Notes:

2024 ROSE STUDY - GENERAL SCORING SHEET

Patient name:

Date & Time:

**6) cVOR + VOR**		
**VOR Cancellation (cVOR)**		
**cVOR**<br>*Mild: Deviation easily corrected*<br>*Severe: Consistent missing of target*	Eyes remain on the target	0
	Mild corrective saccades (under/overshoot)	1
	Severe corrective saccades (under/overshoot)	2

**Vestibular-ocular reflex (VOR)**			
**VOR**		Horizontal	Vertical
	Stable target	0	0
	Blurry or jumping target	1	1
	Inability to see target (E)	2	2
	**Total VOR + cVOR Score**	**/6**	
	**Excessive blinking of the eyes**	(Y) □ (N) □	

Notes:

**TOTAL**			
**Test Item**	**Score**	**VAS**	**What is/are the most provocative**
Baseline		/10	
Smooth Pursuits	/14	/10	
Vergence	/4	/10	
Saccades	/16	/10	
Gaze Fixation + Cover Tests	/12	/10	
cVOR +VOR Score	/6	/10	
Excessive blinking of the eyes<br>*No (Y) = 0<br>*≥ 1 (Y) = 1<br>*≥ 3 (Y) = 2	/2		
**Total Score**	**/50**	**/60**	
**Total Score without Vergence**<br>*Calculate only if patient sees double	**/46**		
