# Supplementary material for: Rehabilitation Oculomotor Screening Evaluation in Persons with Traumatic Brain Injury
Source: J Eye Mov Res. 2026 Jul 2;19(4):70. doi: 10.3390/jemr19040070 (PMC13398043; doi:10.3390/jemr19040070)
Supplement: Supplementary file 1 [file jemr-19-00070-s001.zip › Supplementary File S2_ROSE instructions.pdf]

Rehabilitation Oculomotor Screening Evaluation (ROSE) **INSTRUCTIONS**

Equipment Needed: 30cm ruler with a small circular sticker at each end, metronome (app), stopwatch (app), eye cover (optional), pen light, a paper with a size 12, Calibri, "E" in the centre.

Smooth Pursuit

- Hold a small fixation target 40cm from the subject's nasion.
- Instruct them to look at the target and follow it while it moves.
- The subject should hold their head still. If unable to comply, stabilize their head for testing.
- Slowly (<40°/s) move the target to a span of 45° from midline (~40cm).
- Perform **2 cycles in all three directions** (there & back = 1 cycle). Look for deficits

Movement quality: *Small amplitude: ~2-5mm* *Large amplitude: >5mm*

Amplitude + jerkiness: *No jerkiness/ catch-up saccades OR less than 2 saccades*

Small amplitude OR 3-5 saccades *Large amplitude OR >5 saccades*

Asymmetry: *Symmetrical* *Minor* *Major*

- Count # of saccades, mark location of saccades during smooth pursuits on graph

Vergence

- Begin by holding a **30 cm ruler** on the subject's mid forehead (2cm above nasion).
- Slowly move the target toward the subject's nasion.
- Record the distance when the subject reports seeing double or when one of the eyes shift outwards (whichever occurs first) (**NPC**).

< 5.0cm of the nasion *>5.0cm & <10cm of the nasion* *>10cm of the nasion*

- Slowly move back out and record the distance (cm) when subject reports seeing the target clearly (I.e., no longer double) (**Recovery**).

< 7.0cm of the nasion *>7.0 & <12 cm of the nasion* *>12 cm of the nasion*

- **Repeat 3x trials** **If unable to test (no double vision), remove test from final score.
  Symptom Rating (Smooth Pursuit + Vergence)

Saccades

- The subject will be looking back and forth between the 2 targets on the ruler that is **40cm away** (15cm for vergence).
- Instruct the subject to look at the back and forth in between the two target as fast as possible for **8 seconds** (using a timer).
- Count the number of cycles performed in each plane. # of Saccadic Cycles (there & back = 1 cycle): *>7* *4-7* *<4*

- Do not allow the subject to move their head.
- Look for signs of apraxia, hypometria, dysmetria, inaccuracy, ocular flutter, and overshoot.

Accuracy: *Accurate* *Mild under/overshoot* *Severe under/overshoot*

[Mild: Deviation easily corrected] *[Severe: Consistent missing of target.]*

Symmetry of mvts: *Symmetrical* *Mild asymmetry* *Severe asymmetry*

[Mild: Deviates yet synchronous] *[Severe: deviates without synchrony]*

Symptom Rating (Saccades)

Fixation in 8 Gaze Directions (Can be performed immediately after smooth pursuits)

- The subject is advised to focus on a stable target (examiner's finger) positioned at the 45°(~40cm) in each gaze direction (vertical, horizontal, & diagonally) for at least **4** seconds per direction.
- Ensure the subject's head is stabilized in a fixed position for testing.
- Observe for gaze consistency, instability, loss of fixation, and nystagmus.:
    - *Stable fixation in 8 gaze directions. No signs of gaze induced nystagmus or drift. Able to maintain fixation in end of range gaze position for < 4s.*
    - *Gaze induced nystagmus and/or drift observed in 1 direction.*
    - *Gaze induced nystagmus and/or drift observed in >1 directions or not end of range*

Eye Cover Test

- Ask the subject to keep looking at the examiner's nose.
- Cover one eye for **2s**.
- Note if there is any corrective shift (eye mvt) observed in the uncovered eye. Mvt:

remains on target (no correction) *outwards inwards* *upwards downwards*

Alternate Cover-Uncover Test

- Ask the subject to look at the examiner's nose.
- Cover one eye for at least **1s** before switching.
- Once an eye is occluded, examiner observes the eye that was previously covered.
- Note the direction of any movement made on the image below. Uncovered eye mvt:

remain on the target *mainly outwards inwards* *upwards downwards*

- Perform at least **4x/eye**.

Symptom Rating (Cover Tests + Gaze fixation)

VOR Cancellation (cVOR)

- Ask the subject to sit with their feet on the floor, with their arms extended & hands clasped (thumbs up).
- While maintaining focus on the thumb, the examiner will rotate the subject's body, as a whole, achieving a total of 80° rotation.
- Use a **metronome set at 50bpm** (1 beat/direction).
- **Complete 5 cycles** CVOR eye mvt: (corrective saccades)

Remain on the target *Mild (under/overshoot)* *Severe (under/overshoot)*

[Mild CS: Deviation easily corrected] *[Severe CS: Consistent missing of target]*

Vestibular ocular reflex (VOR)

- Ask the subject to fixate at a small target (Calibri, size 12, "E", at the centre of a cue card) placed on a table in front of them (20 of neck flexion).
- Move the subject's head from side to side (i.e., **"no"**), and then up and down (i.e., "**yes"**) for **5 cycles**, while maintaining fixation.
- Provide the cue "**don't resist me**," if the subject is tensing the neck.
- Move the head at **120bpm** (1 beat/cycle), through a range of ±25-30°

VOR: Stable target *Blurry or jumping target* *Inability to see target (E)*

Symptom Rating (cVOR+VOR)

TOTAL: score (/48) + VAS (/50 = 1-X5 (includes Baseline))
Smooth Pursuits (/14), Saccades (/16), Cover Test + Gaze fixation (/12), VOR +cVOR (/6)
What is/are the most provocative
